# Crystal structure, Hirshfeld surface and crystal void analysis, inter­molecular inter­action energies, DFT calculations and energy frameworks of 2*H*-benzo[*b*][1,4]thia­zin-3(4*H*)-one 1,1-dioxide

**DOI:** 10.1107/S205698902300868X

**Published:** 2023-10-19

**Authors:** Ezaddine Irrou, Younesse Ait Elmachkouri, Ahmed Mazzah, Tuncer Hökelek, Amal Haoudi, Joel T. Mague, Mohamed Labd Taha, Nada Kheira Sebbar

**Affiliations:** a Laboratory of Organic and Physical Chemistry, Applied Bioorganic Chemistry Team, Faculty of Sciences, Ibn Zohr University, Agadir, Morocco; b University of Lille, CNRS, UAR 3290, MSAP, Miniaturization for Synthesis, Analysis and Proteomics, F-59000 Lille, France; c Department of Physics, Hacettepe University, 06800 Beytepe, Ankara, Türkiye; d Laboratory of Applied Organic Chemistry, Faculty of Science and Technology, University of Sidi Mohamed Ben Abdellah BP 2202, Fez, Morocco; e Department of Chemistry, Tulane University, New Orleans, LA 70118, USA; f Laboratory of Heterocyclic Organic Chemistry, Medicines Science Research Center, Pharmacochemistry Competence Center, Mohammed V University in Rabat, Faculté des Sciences, Av. Ibn Battouta, BP 1014, Rabat, Morocco; Vienna University of Technology, Austria

**Keywords:** Crystal structure, hydrogen bond, C—H⋯π(ring) inter­action, π-stacking, sulfone, crystal structure

## Abstract

In the title mol­ecule, C_8_H_7_NO_3_S, the nitro­gen atom has a planar environment, and the thia­zine ring exhibits a screw-boat conformation. In the crystal, corrugated layers of mol­ecules parallel to the *ab* plane are formed by N—H⋯O and C—H⋯O hydrogen bonds together with C—H⋯π(ring) and S=O⋯π(ring) inter­actions. The layers are connected by additional C—H⋯O hydrogen bonds and π-stacking inter­actions. Hirshfeld surface analysis indicates that the most important contributions for the crystal packing are from H⋯O/O⋯H (49.4%), H⋯H (23.0%) and H⋯C/C⋯H (14.1%) inter­actions. The volume of the crystal voids and the percentage of free space were calculated as 75.4 Å^3^ and 9.3%. Density functional theory (DFT) computations revealed N—H⋯O and C—H⋯O hydrogen-bonding energies of 43.3, 34.7 and 34.4 kJ mol^−1^, respectively. Evaluation of the electrostatic, dispersion and total energy frameworks indicate that the stabilization is dominated *via* the electrostatic energy contribution. Moreover, the DFT-optimized structure at the B3LYP/ 6–311 G(d,p) level is compared with the experimentally determined mol­ecular structure in the solid state. The HOMO–LUMO behaviour was elucidated to determine the energy gap.

## Abstract 2

In the title compound, the thia­zine ring exhibits a screw-boat conformation. In the crystal, corrugated layers of mol­ecules parallel to the *ab* plane are formed by N—H⋯O and C—H⋯O hydrogen bonds together with C—H⋯π(ring) and S=O⋯π(ring) inter­actions. The layers are connected by additional C—H⋯O hydrogen bonds and π-stacking inter­actions.

## 1. Chemical context

Numerous heterocyclic compounds containing sulfur and nitro­gen have been extensively studied because of their various biological applications (Gowda *et al.*, 2011 ▸; Sebbar *et al.*, 2020*a* ▸; Fringuelli *et al.*, 2005 ▸). In this respect, 1,4-benzo­thia­zine derivatives possess various pharmacological properties and have therapeutic applications such as anti­fungal (Kamila *et al.*, 2006 ▸), anti-inflammatory (Gowda *et al.*, 2011 ▸), antagonistic (Corelli *et al.*, 1997 ▸), anti-tumour (Abbas & Farghaly, 2010 ▸), anti­oxidant (Bakavoli *et al.*, 2008 ▸), anti­pyretic (Warren & Knaus, 1987 ▸), anti­hypertensive (Fringuelli *et al.*, 2005 ▸) or anti­bacterial effects (Sebbar *et al.*, 2016 ▸, 2020*a* ▸).

Continuing our research on the development of new 1,4-benzo­thia­zine derivatives with potential pharmacological applications, we carried out the oxidation of 3,4-di­hydro-2*H*-1,4-benzo­thia­zin-3-one by potassium permanganate in order to obtain 2*H*-benzo[*b*][1,4]thia­zin-3(4*H*)-one 1,1-dioxide (I) with good yield. We report herein the mol­ecular and crystal structure of this compound, as well as Hirshfeld surface analysis and DFT-computational studies carried out at the B3LYP/6–31 G(d,p) and B3LYP/6–311 G(d,p) levels. 
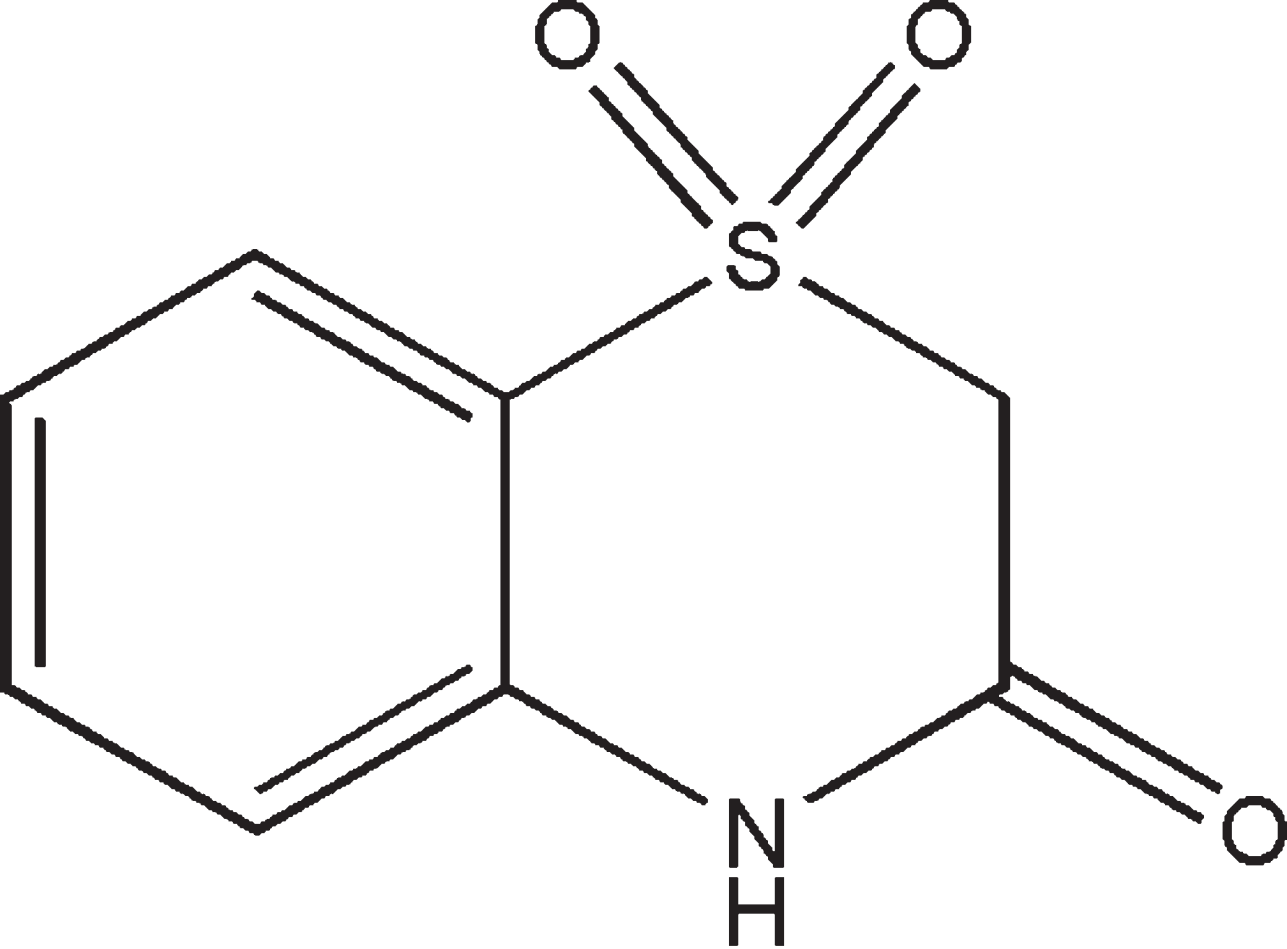


## 2. Structural commentary

**Figure 1:**
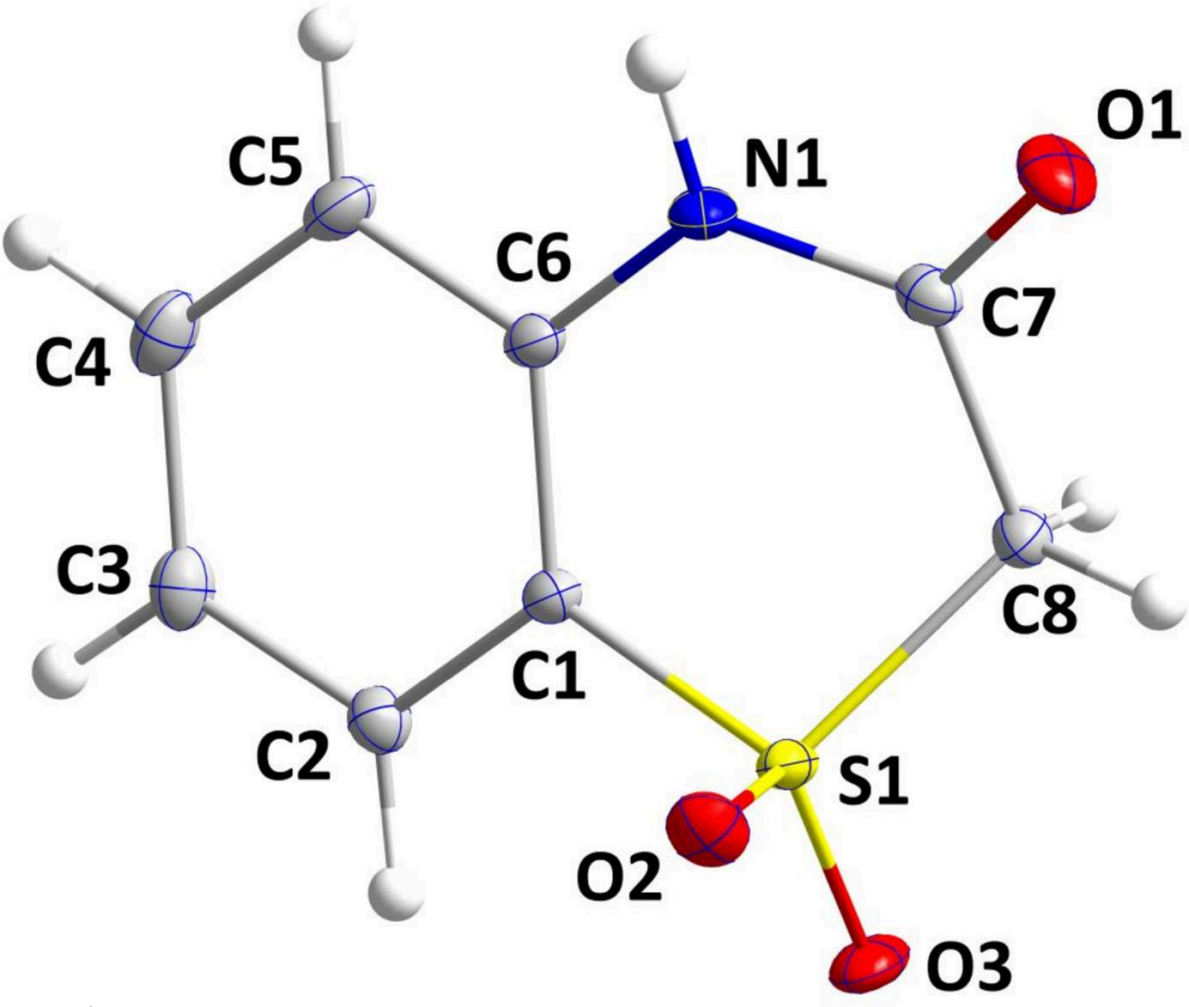
The title mol­ecule with atom labelling and displacement ellipsoids drawn at the 50% probability level.

A puckering analysis (Cremer & Pople, 1975 ▸) of the thia­zine ring (C1, C6, N1, C7, C8, S1) gave the parameters *Q* = 0.5138 (6) Å, θ = 60.49 (7)° and φ = 326.72 (8)°. The distorted screw-boat conformation places O2 in an axial position and O3 in a pseudo-equatorial position (Fig. 1 ▸). The angles about N1 sum up to 360° within experimental error, indicating involvement of the lone pair in the C–N bond. This is reflected in the N1—C7 and N1—C6 distances of 1.3661 (9) and 1.4043 (9) Å, respectively.

## 3. Supra­molecular features

**Figure 2:**
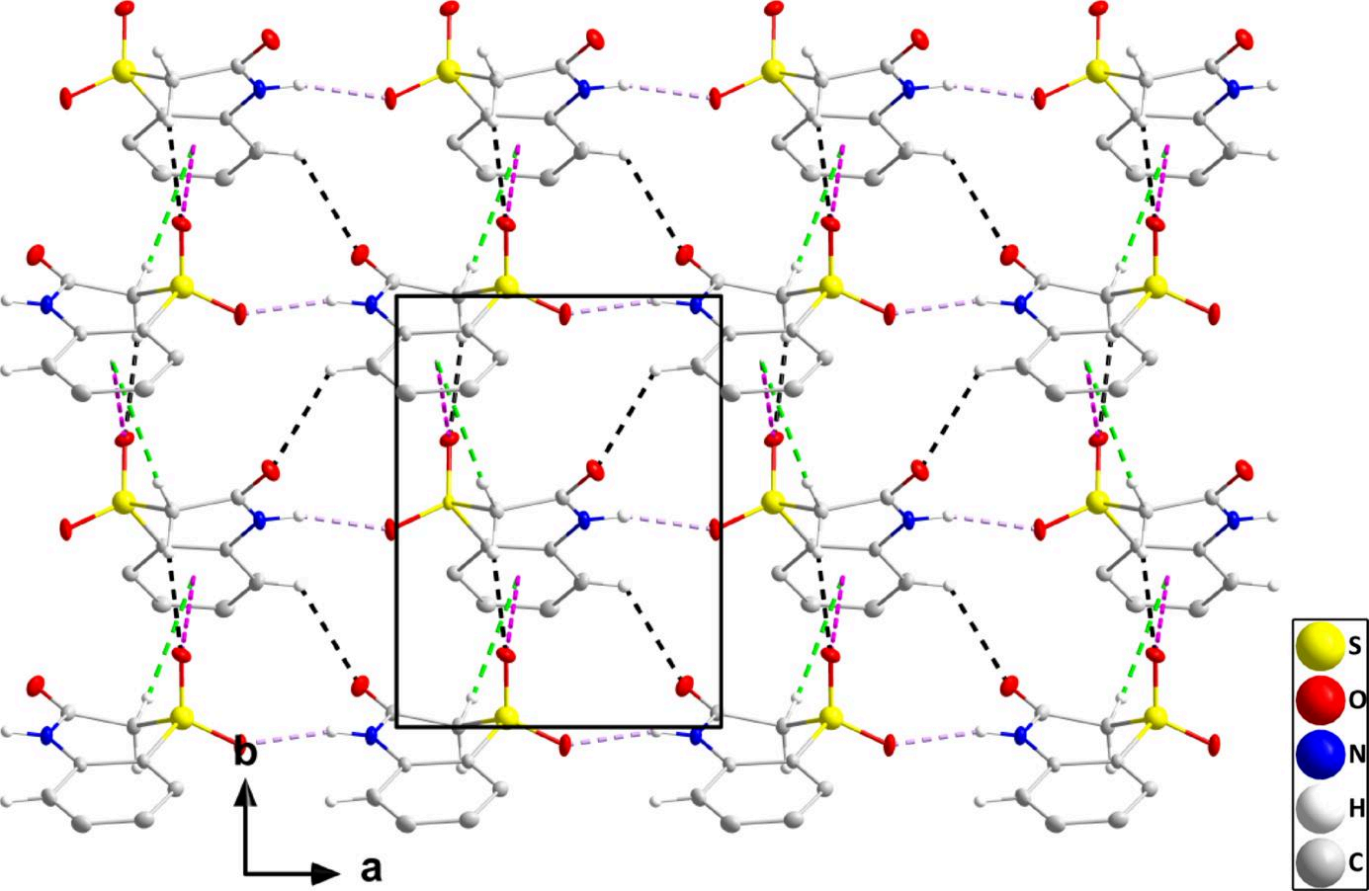
The crystal structure of (I) viewed along the *c* axis with N—H⋯O and C—H⋯O hydrogen bonds depicted, respectively, by violet and black dashed lines. C—H⋯π(ring) and C=O⋯π(ring) inter­actions are depicted, respectively, by green and dark-pink dashed lines and non-inter­acting hydrogen atoms are omitted for clarity.

**Figure 3:**
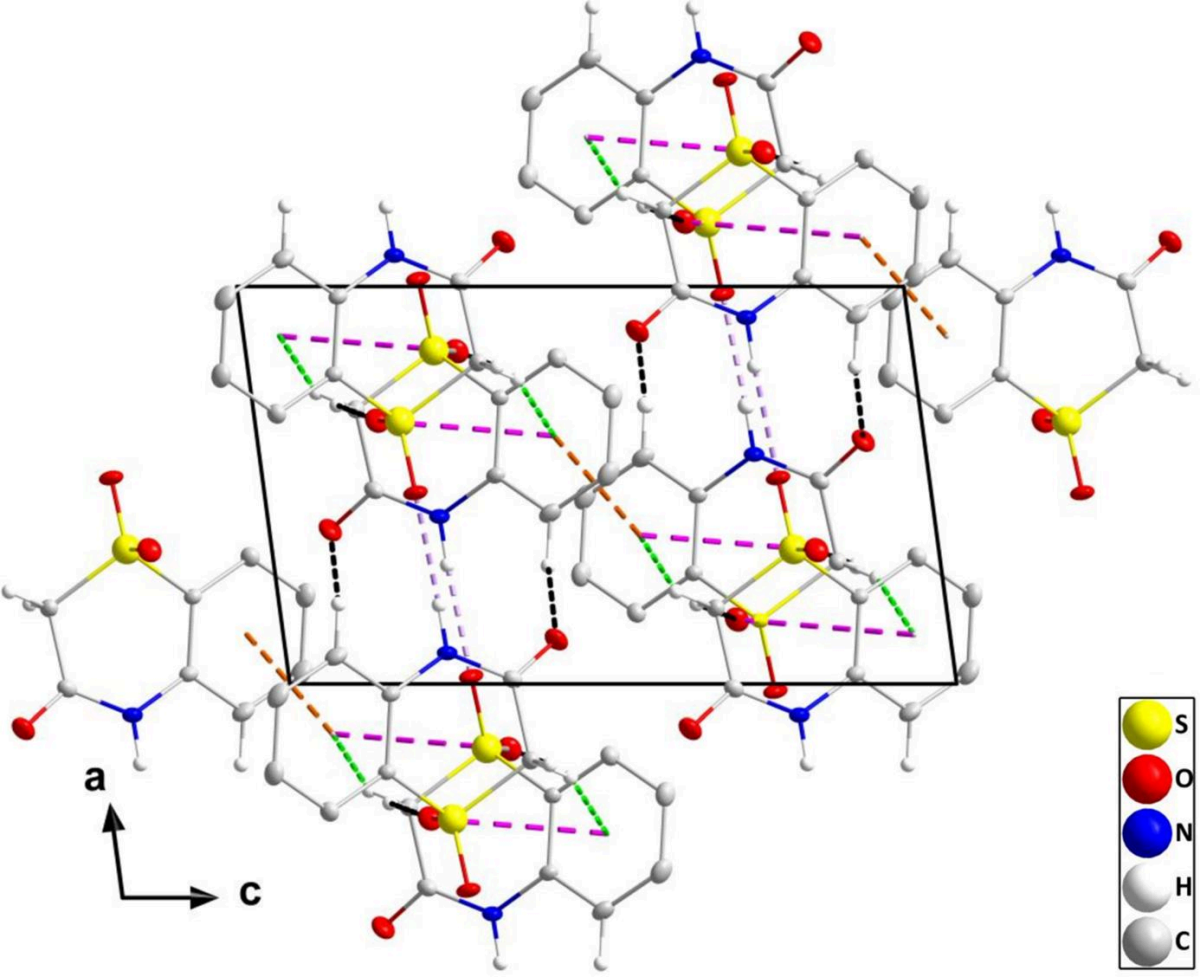
The crystal structure of (I) viewed along the *b* axis with N—H⋯O and C—H⋯O hydrogen bonds depicted, respectively, by violet and black dashed lines. C—H⋯π(ring), C=O⋯π(ring) and slipped π-stacking inter­actions are depicted, respectively, by green, dark-pink and orange dashed lines. Non-inter­acting hydrogen atoms are omitted for clarity.

In the crystal, N1—H1⋯O3 hydrogen bonds (Table 1 ▸) form chains of mol­ecules extending parallel to the *a* axis. These chains are connected into corrugated layers parallel to the *ab* plane by C8—H8*B*⋯O2 hydrogen bonds together with C8—H8*A*⋯*Cg*2 and S1=O2⋯*Cg*2^i^ inter­actions [O2⋯*Cg*2 = 3.6233 (7) Å, S1⋯*Cg*2 = 4.1655 (5) Å, S1=O2⋯*Cg*2^i^ = 101.77 (3)°; symmetry code: (i) −*x* + 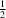 , *y* + 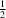 , −*z* + 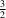 ; Fig. 2 ▸]. The layers are connected by C5—H5⋯O1 hydrogen bonds (Table 1 ▸) and slipped π–π stacking inter­actions between inversion-related C1–C6 rings [*Cg*2⋯*Cg*2(1 −*x*, 1 −*y*, 1 −*z*) = 3.7353 (5) Å, slippage = 1.55 Å] into a tri-periodic network structure (Fig. 3 ▸).

## 4. Hirshfeld surface analysis

**Figure 4:**
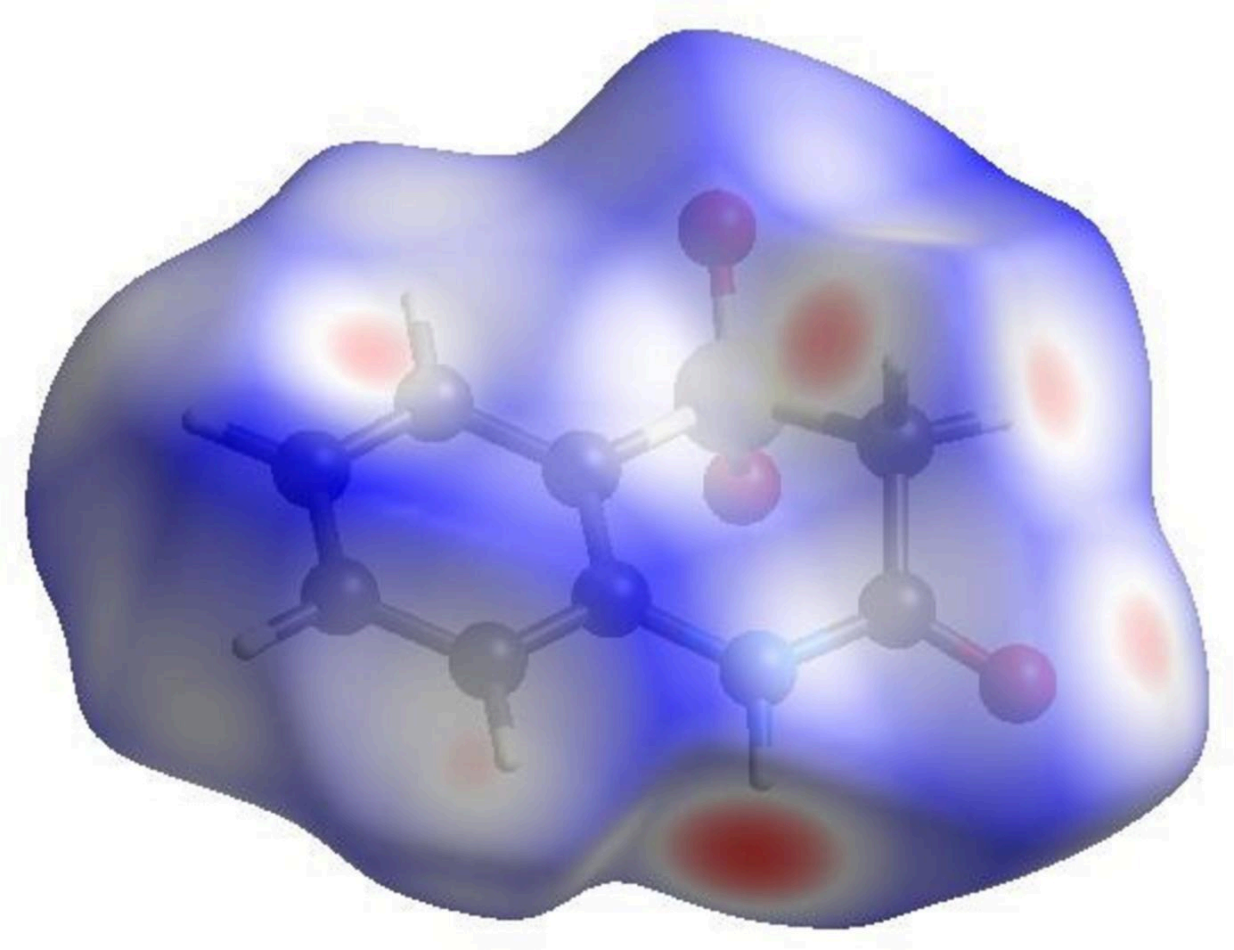
View of the three-dimensional Hirshfeld surface of (I) plotted over *d*_norm_.

**Figure 5:**
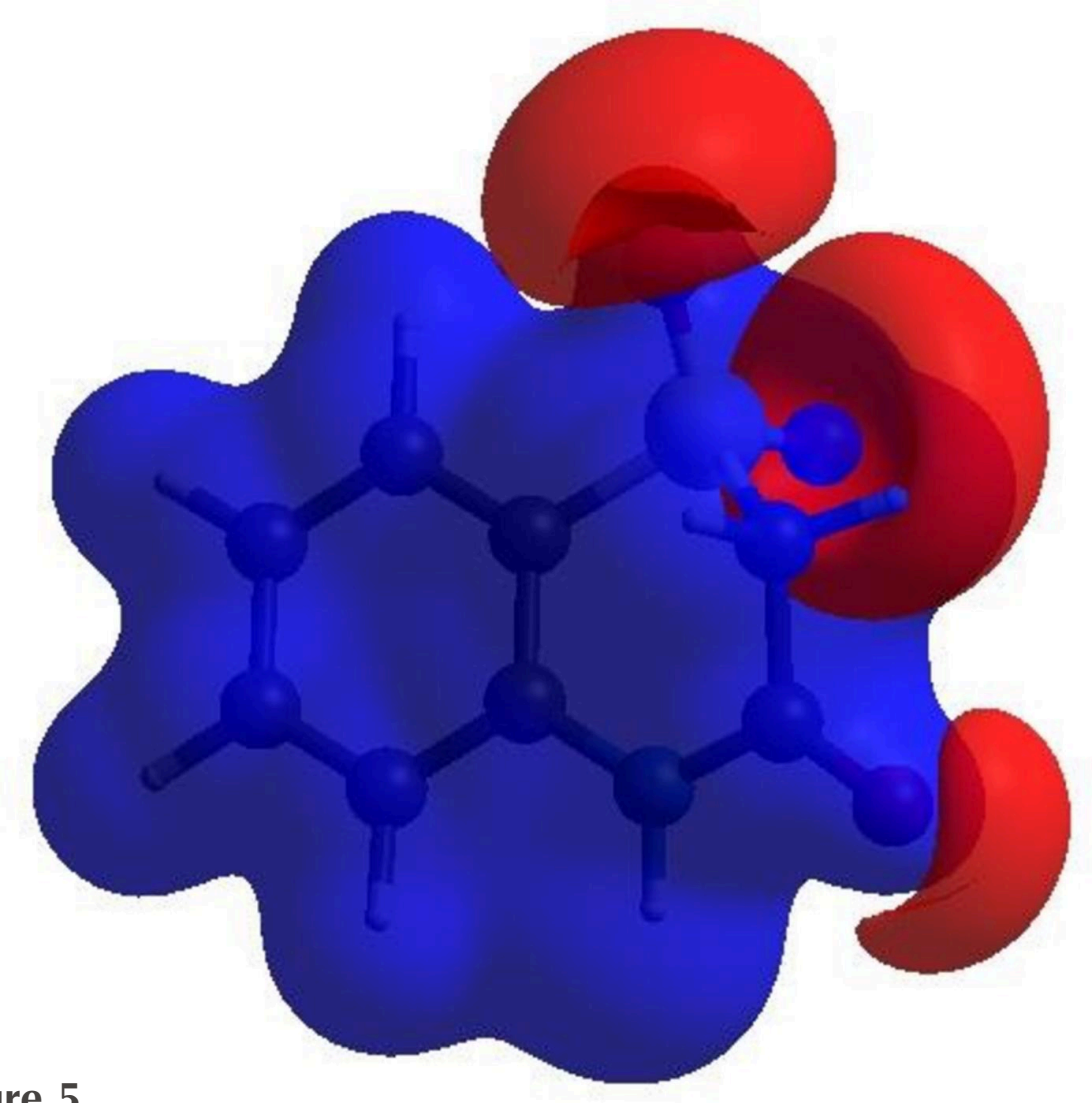
View of the three-dimensional Hirshfeld surface of (I) plotted over electrostatic potential energy using the STO-3 G basis set at the Hartree–Fock level of theory.

**Figure 6:**
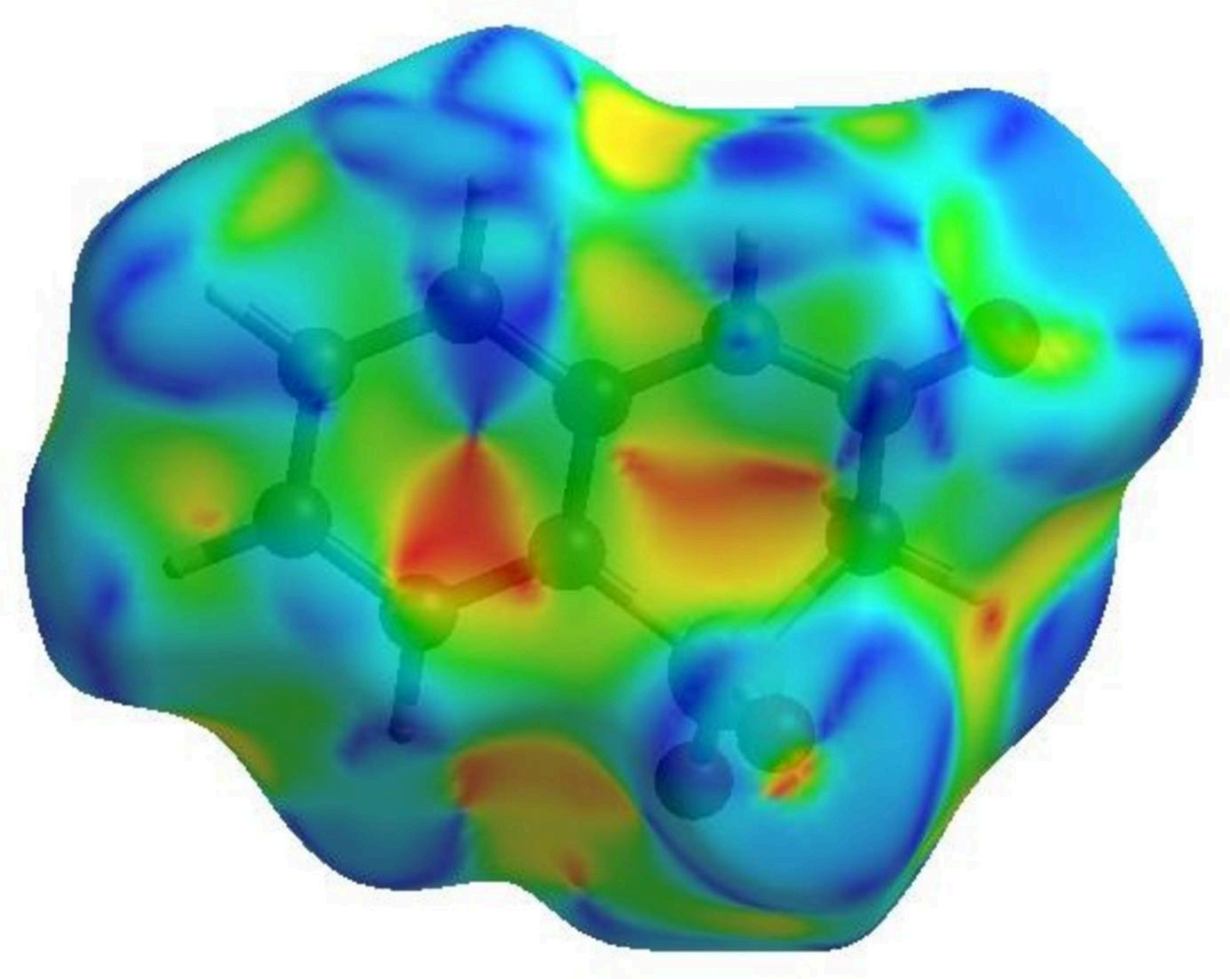
Hirshfeld surface of (I) plotted over shape-index.

**Figure 7:**
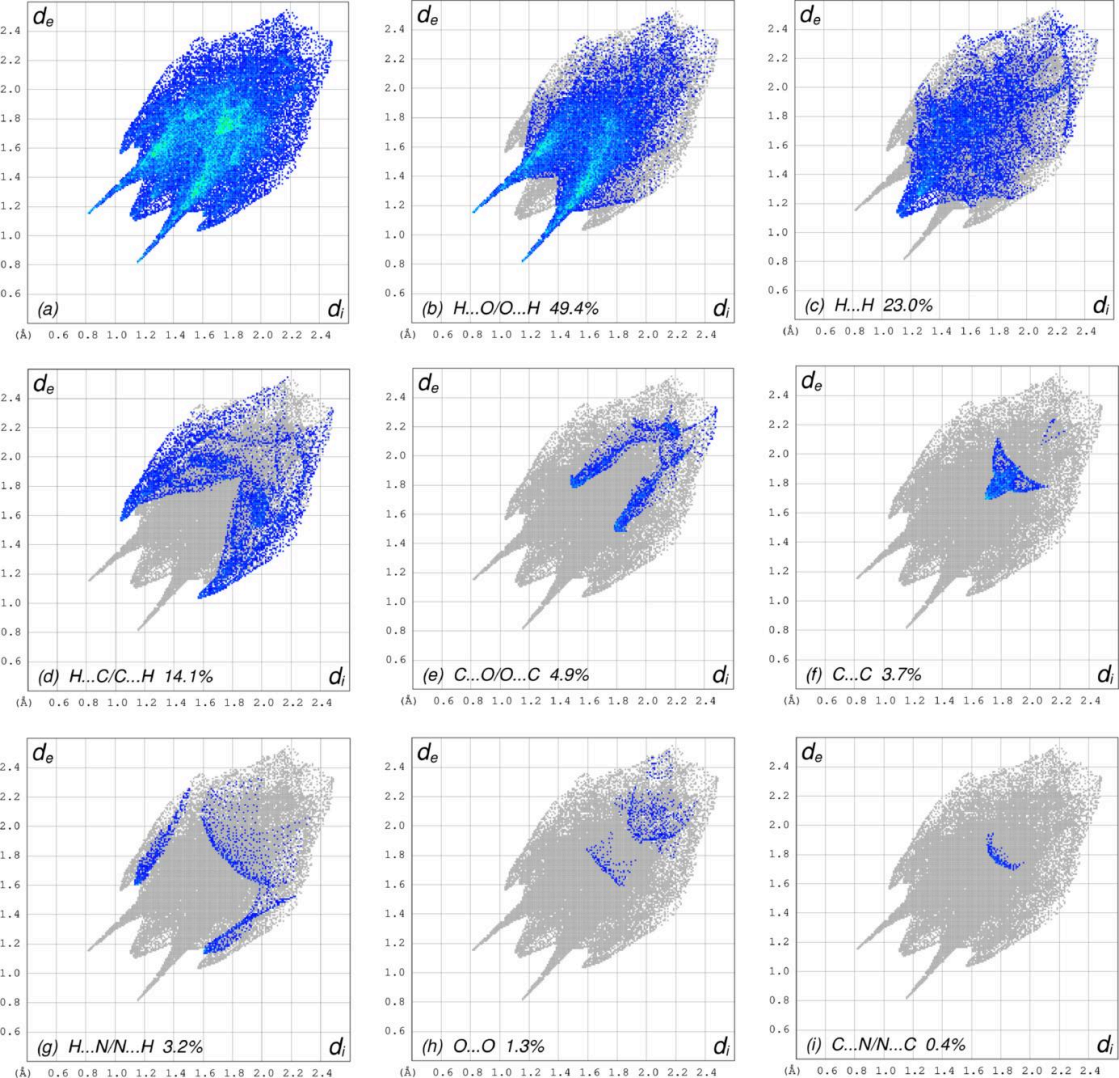
The full two-dimensional fingerprint plots for the title compound, showing (*a*) all inter­actions, (*b*) H⋯O/O⋯H, (*c*) H⋯H, (*d*) H⋯C/C⋯H, (*e*) O⋯C/C⋯O, (*f*) C⋯C, (*g*) H⋯N/N⋯H, (*h*) O⋯O and (*i*) C⋯N/N⋯C inter­actions. The *d*_i_ and *d*_e_ values are the closest inter­nal and external distances (in Å) from given points on the Hirshfeld surface contacts.

**Figure 8:**
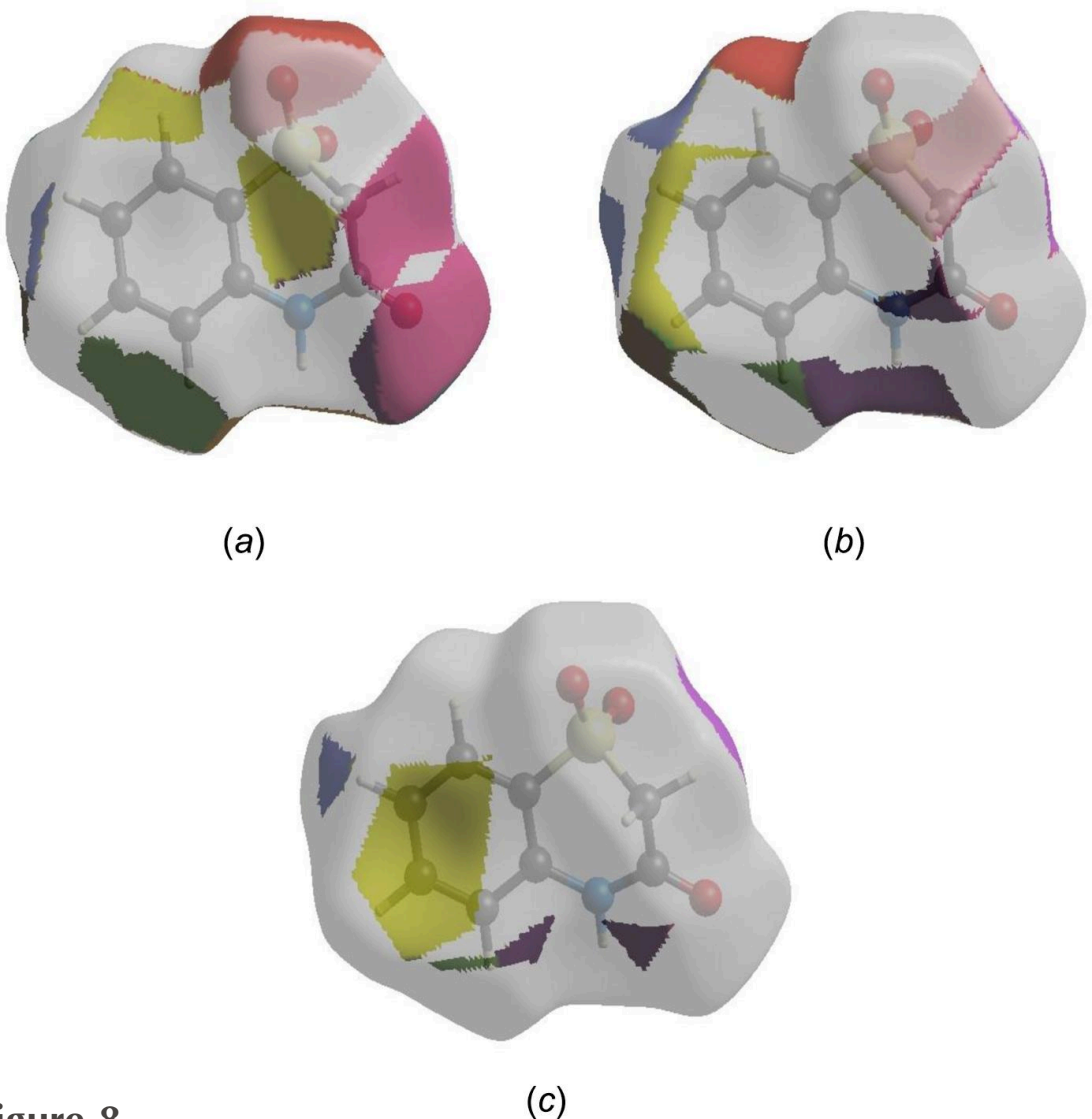
Hirshfeld surface representations of (I) with the function *d*_norm_ plotted onto the surface for (*a*) H⋯O/O⋯H, (*b*) H⋯H and (*c*) H⋯C/C⋯H inter­actions.

To visualize the inter­molecular inter­actions in the crystal of (I), a Hirshfeld surface (HS) analysis (Hirshfeld, 1977 ▸) was carried out with *Crystal Explorer* (Spackman *et al.*, 2021 ▸). In the HS plotted over *d*_norm_ in the range −0.4976 to 1.2253 a.u. (Fig. 4 ▸), the white surface indicates contacts with distances equal to the sum of van der Waals radii and the red and blue colours indicate distances shorter (in close contact) or longer (distant contact) than the van der Waals radii, respectively (Venkatesan *et al.*, 2016 ▸). The bright-red spots indicate their roles as the respective donors and/or acceptors; they also appear as blue and red regions corresponding to positive and negative potentials on the HS mapped over electrostatic potential (Spackman *et al.*, 2008 ▸; Jayatilaka *et al.*, 2005 ▸) in the range −0.05 to 0.05 a.u., as shown in Fig. 5 ▸. The blue regions indicate positive electrostatic potential (hydrogen-bond donors), while the red regions indicate negative electrostatic potential (hydrogen-bond acceptors). The shape-index of the HS is a tool to visualize the π–π stacking by the presence of adjacent red and blue triangles. Fig. 6 ▸ clearly suggests that there are π–π inter­actions in (I). The overall two-dimensional fingerprint plot, Fig. 7 ▸ *a*, and those delineated into H⋯O/O⋯H, H⋯H, H⋯C/C⋯H, C⋯O/O⋯C,C⋯C, H⋯N/N⋯H, O⋯O and C⋯N/N⋯C contacts (McKinnon *et al.*, 2007 ▸) are illustrated in Fig. 7 ▸ *b*–*i*, respectively, together with their relative contributions to the Hirshfeld surface. The most important inter­action is H⋯O/O⋯H, contributing 49.4% to the overall crystal packing, which is reflected in Fig. 7 ▸ *b*, where the symmetric pair of spikes is observed with the tips at *d*_e_ + *d*_i_ = 1.98 Å. The H⋯H contacts contribute 23.0% to the overall crystal packing, which is reflected in Fig. 7 ▸ *c* as widely scattered points of high density due to the large hydrogen content of the mol­ecule with the tip at *d*_e_ = *d*_i_ = 1.13 Å. In the presence of C—H⋯π inter­actions, the pair of characteristic wings in the fingerprint plot delineated into H⋯C/C⋯H contacts, Fig. 7 ▸ *d*, make a 14.1% contribution to the HS and viewed with the tips at *d*_e_ + *d*_i_ = 2.59 Å. The wing pair of C⋯O/O⋯C contacts (Fig. 7 ▸ *e*) with 4.9% contribution to the HS is viewed at *d*_e_ + *d*_i_ = 3.30 Å. The C⋯C contacts (Fig. 7 ▸ *f*) appearing as a bullet-shaped distribution of points make a contribution of 3.7% to the HS with the tip at *d*_e_ = *d*_i_ = 1.70 Å. The spikes of H⋯N/N⋯H contacts (Fig. 7 ▸ *g*) with 3.2% contribution to the HS are viewed at *d*_e_ + *d*_i_ = 2.75 Å. Finally, the O⋯O (Fig. 7 ▸ *h*) and C⋯N/N⋯C (Fig. 7 ▸ *i*) contacts contribute 1.3% and 0.4%, respectively, to the HS. The Hirshfeld surface representations with the function *d*_norm_ plotted onto the surface are shown for the H⋯O/O⋯H, H⋯H and H⋯C/C⋯H inter­actions in Fig. 8 ▸ *a*–*c*, respectively. The Hirshfeld surface analysis confirms the importance of H-atom contacts in establishing the packing. The large number of H⋯O/O⋯H, H⋯H and H⋯C/C⋯H inter­actions suggest that van der Waals inter­actions play the major role in the crystal packing (Hathwar *et al.*, 2015 ▸).

**Figure 9:**
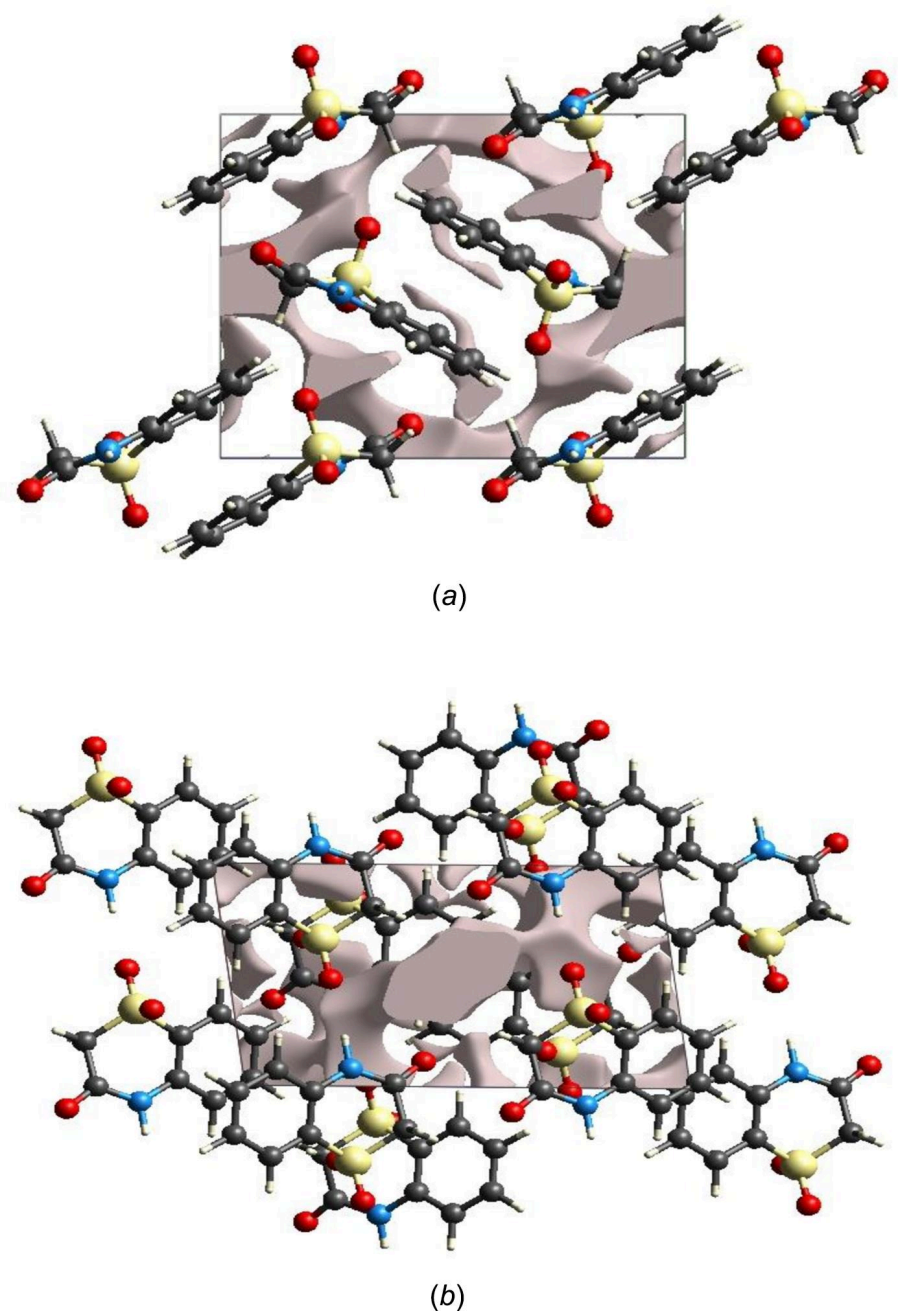
Graphical views of voids in the crystal packing of (I), (*a*) along the *a* axis and (*b*) along the *b* axis.

The strength of the crystal packing is important for determining the response to an applied mechanical force. If the crystal packing results in significant voids, then the mol­ecules are not tightly packed and a small amount of applied external mechanical force may easily break the crystal. To check the mechanical stability of the crystal, a void analysis was performed by adding up the electron densities of the spherically symmetric atoms contained in the asymmetric unit (Turner *et al.*, 2011 ▸). The void surface is defined as an isosurface of the procrystal electron density and is calculated for the whole unit cell where the void surface meets the boundary of the unit cell and capping faces are generated to create an enclosed volume. The volume of the crystal voids (Fig. 9 ▸ *a*,*b*) and the percentage of free space in the unit cell are calculated as 75.4 Å^3^ and 9.3%, respectively. Thus, the crystal packing appears compact and the mechanical stability should be substantial.

## 5. Inter­action energy calculations and energy frameworks

The inter­molecular inter­action energies were calculated using the CEB3LYP/631G(d,p) energy model available in *CrystalExplorer* (Spackman *et al.*, 2021 ▸), where a cluster of mol­ecules is generated by applying crystallographic symmetry operations with respect to a selected central mol­ecule within the radius of 3.8 Å by default (Turner *et al.*, 2014 ▸). The total inter­molecular energy (*E*_tot_) is the sum of electrostatic (*E*_ele_), polarization (*E*_pol_), dispersion (*E*_dis_) and exchange-repulsion (*E*_rep_) energies (Turner *et al.*, 2015 ▸) with scale factors of 1.057, 0.740, 0.871 and 0.618, respectively (Mackenzie *et al.*, 2017 ▸). Hydrogen-bonding inter­action energies (in kJ mol^−1^) were calculated to be [−18.5 (*E*_ele_), −5.2 (*E*_pol_), −41.4 (*E*_dis_), 26.2 (*E*_rep_) and −43.3 (*E*_tot_)] for N1—H1⋯O3, [−22.4 (*E*_ele_), −4.8 (*E*_pol_), −28.3 (*E*_dis_), 27.9 (*E*_rep_) and −34.7 (*E*_tot_)] for C8—H8*B*⋯O2 and [−20.6 (*E*_ele_), −5.8 (*E*_pol_), −24.6 (*E*_dis_), 21.2 (*E*_rep_) and −34.4 (*E*_tot_)] for C5—H5⋯O1.

**Figure 10:**
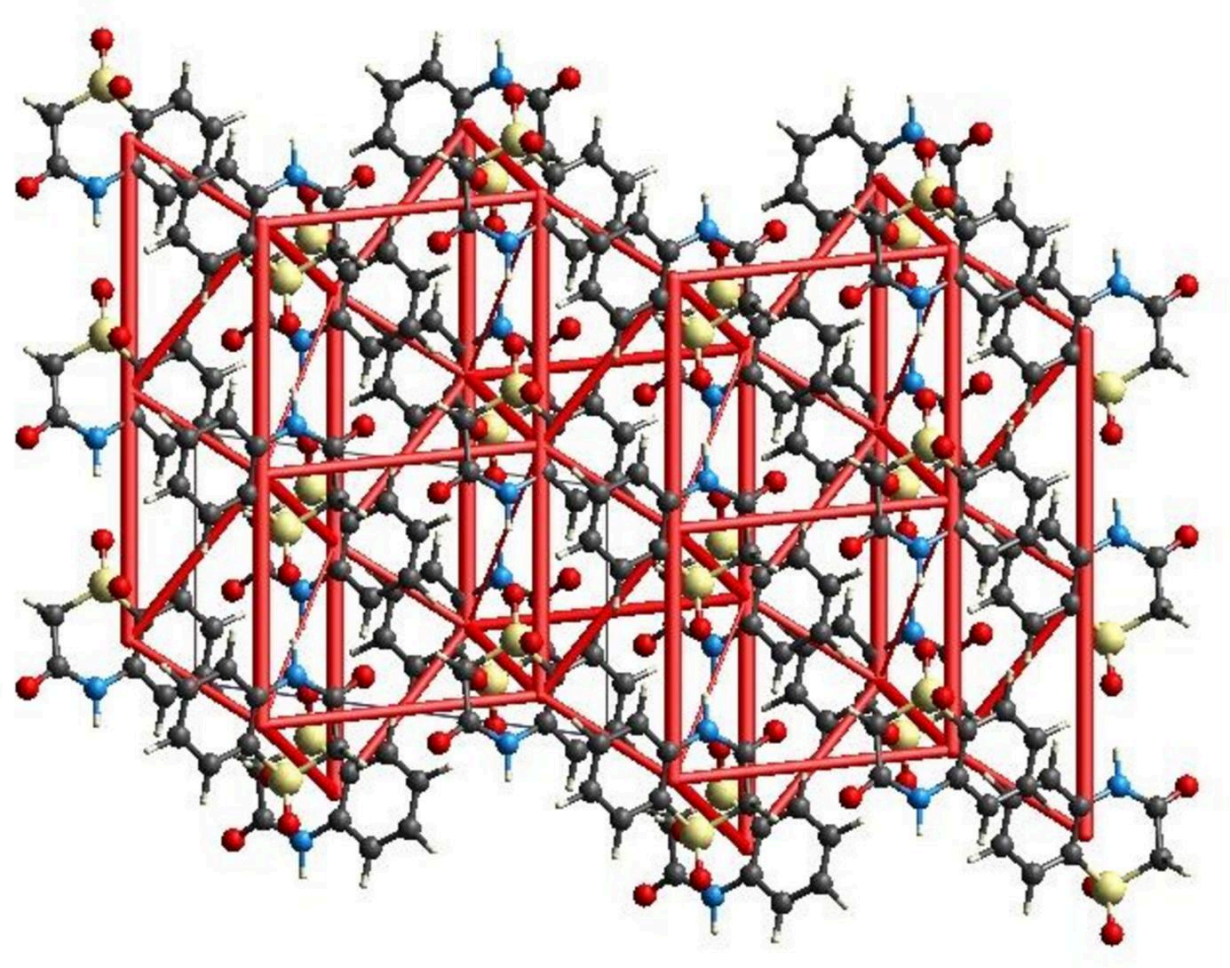
The energy framework for the electrostatic energy, viewed down the *b* axis for a cluster of mol­ecules, where the *a* axis is vertical and the *c* axis is horizontal. The cylindrical radius is proportional to the relative strength of the corresponding energy and adjusted to the scale factor of 80 with a cut-off value of 5 kJ mol^−1^ within 2 × 2 × 2 unit cells.

Energy frameworks combine the calculation of inter­molecular inter­action energies with a graphical representation of their magnitude (Turner *et al.*, 2015 ▸). Energies between mol­ecular pairs are represented as cylinders joining the centroids of pairs of mol­ecules with the cylinder radius proportional to the relative strength of the corresponding inter­action energy. Energy frameworks were constructed for *E*_ele_ (shown in Fig. 10 ▸), *E*_dis_ and *E*_tot_. The evaluation of the electrostatic, dispersion and total energy frameworks indicate that the stabilization is dominated *via* the electrostatic energy contribution in the crystal structure of (I).

## 6. DFT calculations

The optimized structure of (I) was computed in the gas phase using density functional theory (DFT) with the standard B3LYP functional and 6–311 G(d,p) basis-set calculations (Becke, 1993 ▸), employing the *GAUSSIAN 09* software (Frisch *et al.*, 2009 ▸). The theoretical and experimental results exhibit a good agreement, as summarized in Table 2 ▸.

**Figure 11:**
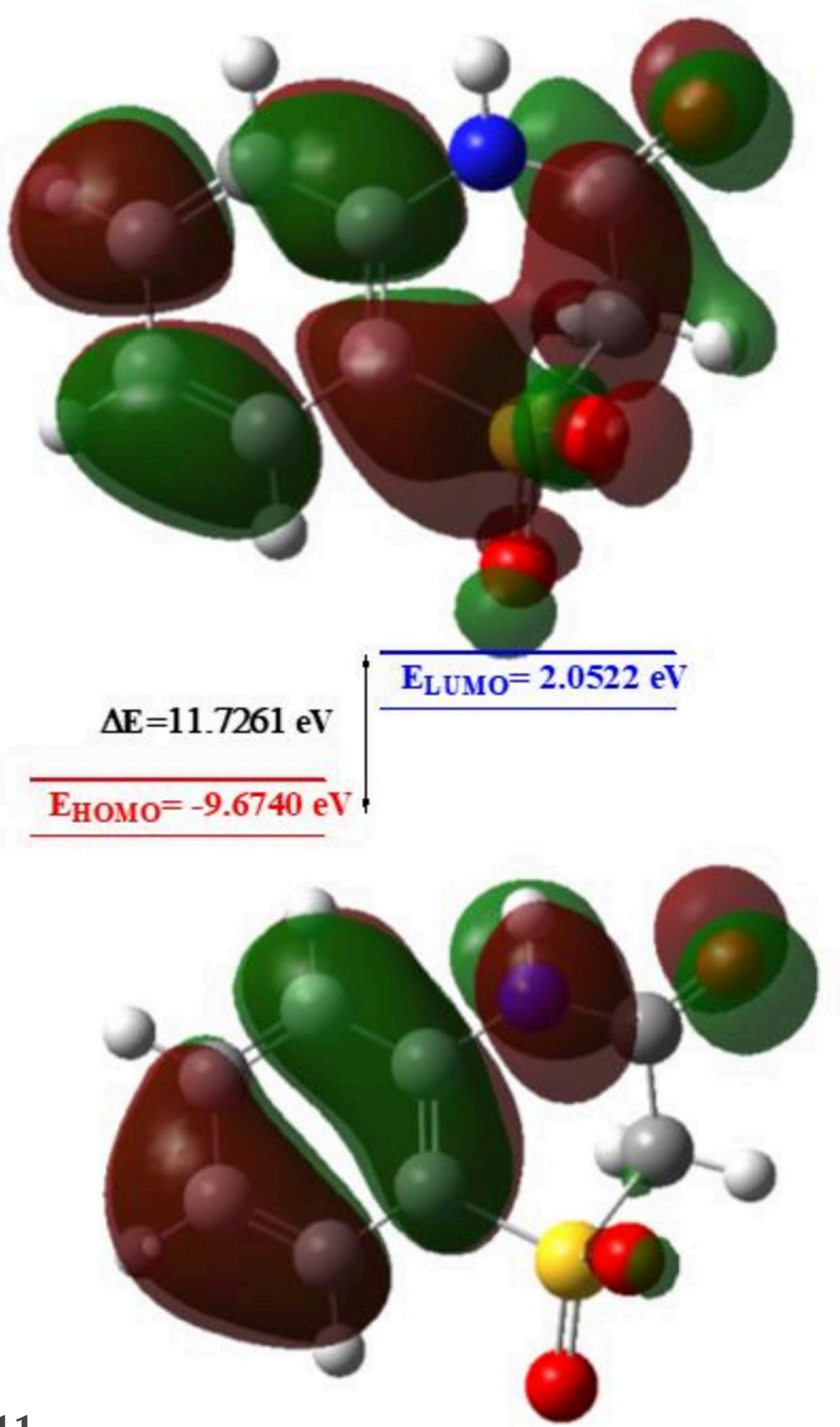
The energy band gap of (I).

The highest-occupied mol­ecular orbital (HOMO), functioning as an electron donor, and the lowest-unoccupied mol­ecular orbital (LUMO), acting as an electron acceptor, serve as vital parameters in quantum chemistry. A small energy gap signifies high mol­ecular polarizability and enhanced chemical reactivity. The DFT calculations provided crucial insights into the reactivity and site selectivity of the mol­ecular framework. Parameters such as *E*_HOMO_ and *E*_LUMO_, electronegativity (*χ*), hardness (*η*), dipole moment (*μ*), electrophilicity (*ω*) and softness (*σ*) are compiled in Table 3 ▸. Both *η* and *σ* are essential for assessing reactivity and stability. The electron transition from HOMO to LUMO energy levels is depicted in Fig. 11 ▸. Notably, both HOMO and LUMO are localized within the plane spanning the entire 2H-benzo[*b*][1,4]thia­zin-3(4*H*)-one 1,1-dioxide ring. The energy band gap [Δ*E* = *E*_LUMO_ − *E*_HOMO_] for the mol­ecule is 11.7261 eV, and the energies of the frontier mol­ecular orbitals, *E*_HOMO_ and *E*_LUMO_, are −9.6740 eV and 2.0522 eV, respectively.

## 7. Database survey

**Figure 12:**
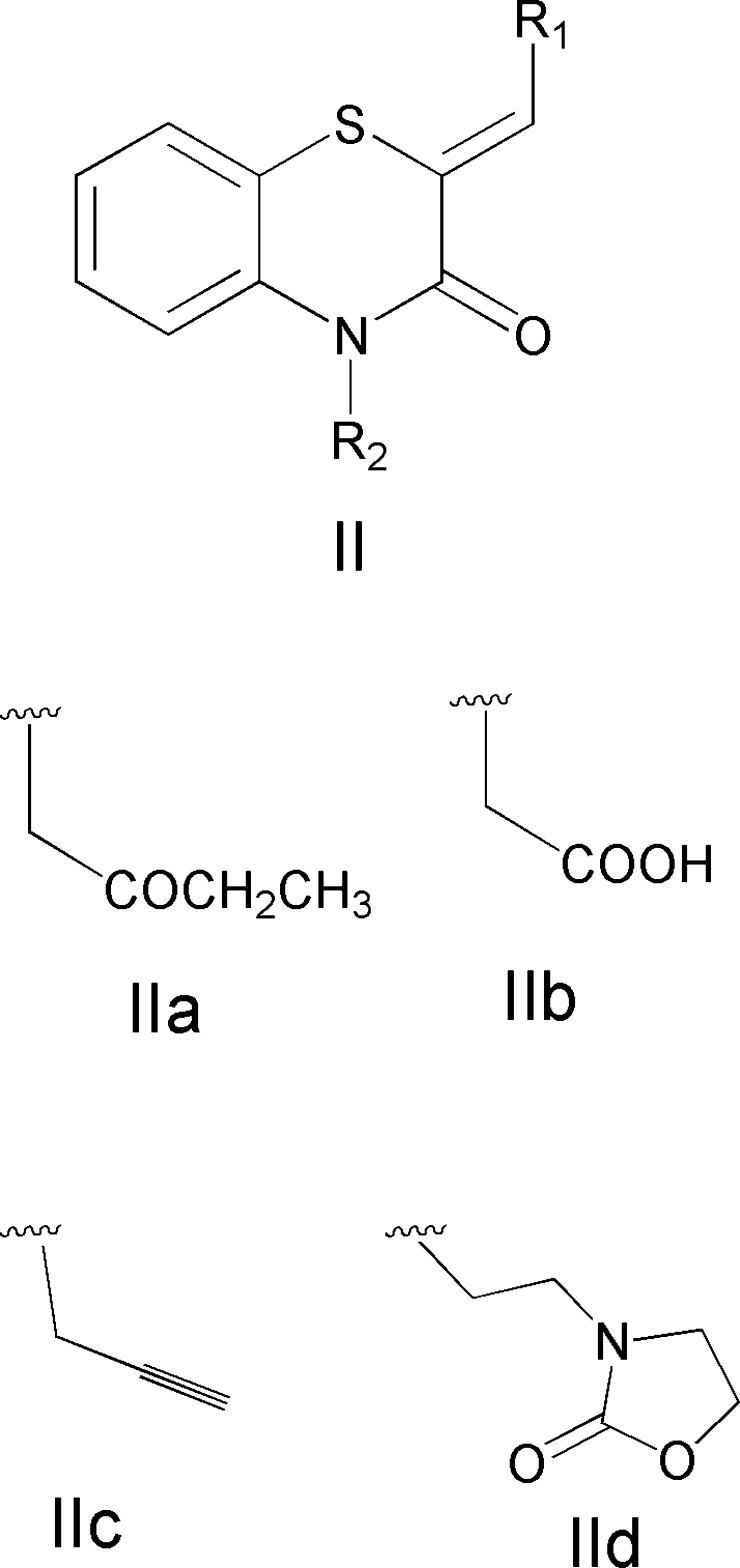
The mol­ecular moieties (II) used for the CSD database search.

A search in the Cambridge Structural Database (CSD, updated March 2023; Groom *et al.*, 2016 ▸) for compounds containing the fragment **II** *(R*1 = Ph or 2-ClC_6_H_4_, *R*2 = C; Fig. 12 ▸), gave 14 hits. With *R*1 = Ph, and with *R*2 = CH_2_COOCH_2_CH_3_ (**IIa**; Sebbar *et al.*, 2020*b* ▸), CH_2_COOH (**IIb**; Sebbar *et al.*, 2016 ▸), CH_2_C≡CH (**IIc**; Sebbar *et al.*, 2014 ▸) and C_5_H_8_NO*2* (**IId**; Sebbar *et al.*, 2016 ▸) (Fig. 12 ▸) are matching candidates. Other examples with *R*1 = 4-FC_6_H_4_ and *R*2 = CH_2_C≡CH (Hni *et al.*, 2019 ▸) and *R*1 = 2-ClC_6_H_4_, *R*2 = CH_2_C≡CH (Sebbar *et al.*, 2017 ▸) are also known.

## 8. Synthesis and crystallization

3,4-Di­hydro-2*H*-1,4-benzo­thia­zin-3-one (1.2 mmol) was dissolved in 3 ml of acetic acid and added dropwise into a solution of potassium permanganate (1.81 mmol) in 6 ml of water. After stirring for one h at room temperature, a solution of sodium thio­sulfate penta­hydrate (20%_wt_) was added to react with excessive potassium permanganate. The precipitate obtained was filtered and recrystallized from ethanol to yield single-crystals suitable for X-ray structure analysis..

## 9. Refinement

Crystal data, data collection and structure refinement details are summarized in Table 4 ▸. H-atoms attached to carbon were placed in calculated positions (C—H = 0.95–0.99 Å) and were included as riding contributions with isotropic displacement parameters 1.2 or 1.5 times those of the attached atoms. That attached to nitro­gen was placed in a location derived from a difference map and refined with a DFIX 0.91 0.01 instruction. Two reflections affected by the beamstop were omitted from the final refinement.

## Supplementary Material

Crystal structure: contains datablock(s) global, I. DOI: 10.1107/S205698902300868X/wm5698sup1.cif

Structure factors: contains datablock(s) I. DOI: 10.1107/S205698902300868X/wm5698Isup2.hkl

Supporting information file. DOI: 10.1107/S205698902300868X/wm5698Isup3.cdx

Supporting information file. DOI: 10.1107/S205698902300868X/wm5698Isup4.cml

CCDC reference: 2298958

Additional supporting information:  crystallographic information; 3D view; checkCIF report

## Figures and Tables

**Table 1:** Hydrogen-bond geometry (Å, °) *Cg*2 is the centroid of the C1–C6 benzene ring.

*D*—H⋯*A*	*D*—H	H⋯*A*	*D*⋯*A*	*D*—H⋯*A*
N1—H1⋯O3^i^	0.89 (1)	2.08 (1)	2.9472 (8)	165 (1)
C5—H5⋯O1^ii^	0.95	2.58	3.2330 (9)	126
C8—H8*A*⋯*Cg*2^iii^	0.99	2.93	3.7933 (8)	146
C8—H8*B*⋯O2^iv^	0.99	2.39	3.2126 (9)	140

Symmetry codes: (i) 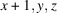 ; (ii) 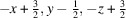 ; (iii) 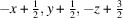 ; (iv) 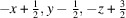 .

**Table 2:** Comparison of the selected (X-ray and DFT) geometric data (Å, °)

Bonds/angles	X-ray	B3LYP/6–311G(d,p)
S1—O2	1.4450 (6)	1.50996
S1—O3	1.4464 (6)	1.59088
S1—C1	1.7478 (6)	1.78874
S1—C8	1.7649 (7)	1.80529
O1—C7	1.2203 (8)	1.21656
N1—C7	1.3661 (9)	1.37417
N1—C6	1.4043 (9)	1.39867
O2—S1—O3	117.64 (4)	118.09813
O2—S1—C1	109.21 (3)	109.40063
O3—S1—C1	109.56 (3)	109.84640
O2—S1—C8	108.59 (3)	109.10007
O3—S1—C8	109.59 (3)	109.62080
C1—S1—C8	100.95 (3)	99.96775
C7—N1—C6	127.24 (6)	127.88849
C7—N1—H1	116.1 (10)	115.98354

**Table 3:** Calculated energies

Mol­ecular Energy (a.u.) (eV)	Compound (I)
Total Energy, *TE* (eV)	−26615,8936
*E*_HOMO_ (eV)	−9.6740
*E*_LUMO_ (eV)	2.0522
Gap, *ΔE* (eV)	11.7261
Dipole moment, *μ* (Debye)	7.583751
Ionization potential, *I* (eV)	9.6740
Electron affinity, *A*	2.0522
Electronegativity, *χ*	−3.8109
Hardness, *η*	−5.8631
Electrophilicity index, *ω*	−1.2385
Softness *σ*,	−0.1706
Fraction of electron transferred, *ΔN*	−0.9219

**Table 4:** Experimental details

Crystal data	
Chemical formula	C_8_H_7_NO_3_S
*M*_r_	197.21
Crystal system, space group	Monoclinic, *P*2_1_/*n*
Temperature (K)	125
*a*, *b*, *c* (Å)	7.2179 (6), 9.5043 (8), 11.9945 (9)
β (°)	97.584 (2)
*V* (Å^3^)	815.64 (11)
*Z*	4
Radiation type	Mo *K*α
μ (mm^−1^)	0.37
Crystal size (mm)	0.39 × 0.21 × 0.16

Data collection	
Diffractometer	Bruker D8 QUEST PHOTON 3 diffractometer
Absorption correction	Multi-scan (*SADABS*; Krause *et al.*, 2015 ▸)
*T*_min_, *T*_max_	0.91, 0.94
No. of measured, independent and observed [*I* > 2σ(*I*)] reflections	47688, 3957, 3739
*R*_int_	0.027
(sin θ/λ)_max_ (Å^−1^)	0.836

Refinement	
*R*[*F* ^2^ > 2σ(*F* ^2^)], *wR*(*F* ^2^), *S*	0.025, 0.074, 1.04
No. of reflections	3957
No. of parameters	122
No. of restraints	1
H-atom treatment	H atoms treated by a mixture of independent and constrained refinement
Δρ_max_, Δρ_min_ (e Å^−3^)	0.52, −0.36

Computer programs: *APEX4* and *SAINT* (Bruker, 2021 ▸), *SHELXT* (Sheldrick, 2015*a* ▸), *SHELXL2018/1* (Sheldrick, 2015*b* ▸), *DIAMOND* (Brandenburg & Putz, 2012 ▸) and *SHELXTL* (Sheldrick, 2008 ▸).

## supplementary crystallographic information

### Crystal data

**Table:**

C_8_H_7_NO_3_S	*F*(000) = 408
*M**_r_* = 197.21	*D*_x_ = 1.606 Mg m^−^^3^
Monoclinic, *P*2_1_/*n*	Mo *K*α radiation, λ = 0.71073 Å
*a* = 7.2179 (6) Å	Cell parameters from 9215 reflections
*b* = 9.5043 (8) Å	θ = 2.9–36.4°
*c* = 11.9945 (9) Å	µ = 0.37 mm^−^^1^
β = 97.584 (2)°	*T* = 125 K
*V* = 815.64 (11) Å^3^	Prism, colourless
*Z* = 4	0.39 × 0.21 × 0.16 mm

### Data collection

**Table:**

Bruker D8 QUEST PHOTON 3 diffractometer	3957 independent reflections
Radiation source: fine-focus sealed tube	3739 reflections with *I* > 2σ(*I*)
Graphite monochromator	*R*_int_ = 0.027
Detector resolution: 7.3910 pixels mm^-1^	θ_max_ = 36.4°, θ_min_ = 3.6°
φ and ω scans	*h* = −12→12
Absorption correction: multi-scan (*SADABS*; Krause *et al.*, 2015)	*k* = −15→15
*T*_min_ = 0.91, *T*_max_ = 0.94	*l* = −19→20
47688 measured reflections	

### Refinement

**Table:**

Refinement on *F*^2^	Primary atom site location: dual
Least-squares matrix: full	Secondary atom site location: difference Fourier map
*R*[*F*^2^ > 2σ(*F*^2^)] = 0.025	Hydrogen site location: mixed
*wR*(*F*^2^) = 0.074	H atoms treated by a mixture of independent and constrained refinement
*S* = 1.04	*w* = 1/[σ^2^(*F*_o_^2^) + (0.0383*P*)^2^ + 0.2724*P*] where *P* = (*F*_o_^2^ + 2*F*_c_^2^)/3
3957 reflections	(Δ/σ)_max_ = 0.001
122 parameters	Δρ_max_ = 0.52 e Å^−^^3^
1 restraint	Δρ_min_ = −0.36 e Å^−^^3^

### Special details

**Table:**

Experimental. The diffraction data were obtained from 9 sets of frames, each of width 0.5° in ω or φ, collected with scan parameters determined by the "strategy" routine in *APEX4*. The scan time was 15 sec/frame.
Geometry. All esds (except the esd in the dihedral angle between two l.s. planes) are estimated using the full covariance matrix. The cell esds are taken into account individually in the estimation of esds in distances, angles and torsion angles; correlations between esds in cell parameters are only used when they are defined by crystal symmetry. An approximate (isotropic) treatment of cell esds is used for estimating esds involving l.s. planes.
Refinement. Refinement of F^2^ against ALL reflections. The weighted R-factor wR and goodness of fit S are based on F^2^, conventional R-factors R are based on F, with F set to zero for negative F^2^. The threshold expression of F^2^ > 2sigma(F^2^) is used only for calculating R-factors(gt) etc. and is not relevant to the choice of reflections for refinement. R-factors based on F^2^ are statistically about twice as large as those based on F, and R- factors based on ALL data will be even larger.

### Fractional atomic coordinates and isotropic or equivalent isotropic displacement parameters (Å^2^)

**Table:**

	*x*	*y*	*z*	*U*_iso_*/*U*_eq_	
S1	0.16068 (2)	0.52143 (2)	0.71754 (2)	0.01099 (4)	
O1	0.61228 (8)	0.59530 (6)	0.90859 (5)	0.01991 (10)	
O2	0.16268 (8)	0.66721 (6)	0.68352 (5)	0.01797 (10)	
O3	−0.01882 (7)	0.45794 (7)	0.72667 (5)	0.01832 (10)	
N1	0.57806 (8)	0.47975 (6)	0.74211 (5)	0.01411 (10)	
H1	0.7001 (12)	0.4900 (15)	0.7398 (13)	0.027 (3)*	
C1	0.27959 (8)	0.42044 (6)	0.62800 (5)	0.01059 (9)	
C2	0.17997 (9)	0.35615 (7)	0.53412 (5)	0.01342 (10)	
H2	0.047397	0.361237	0.521776	0.016*	
C3	0.27651 (11)	0.28460 (7)	0.45882 (6)	0.01663 (11)	
H3	0.210847	0.242727	0.393292	0.020*	
C4	0.47092 (11)	0.27481 (8)	0.48036 (6)	0.01841 (12)	
H4	0.536825	0.224837	0.429380	0.022*	
C5	0.56985 (10)	0.33681 (8)	0.57499 (6)	0.01630 (11)	
H5	0.701938	0.327605	0.588942	0.020*	
C6	0.47486 (8)	0.41274 (7)	0.64966 (5)	0.01164 (10)	
C7	0.51172 (9)	0.53288 (7)	0.83499 (5)	0.01315 (10)	
C8	0.30863 (9)	0.50214 (7)	0.84603 (5)	0.01299 (10)	
H8A	0.266578	0.566751	0.902374	0.016*	
H8B	0.298167	0.404753	0.873825	0.016*	

### Atomic displacement parameters (Å^2^)

**Table:**

	*U*^11^	*U*^22^	*U*^33^	*U*^12^	*U*^13^	*U*^23^
S1	0.00776 (7)	0.01295 (7)	0.01219 (7)	0.00109 (4)	0.00109 (4)	−0.00153 (4)
O1	0.0173 (2)	0.0236 (3)	0.0174 (2)	−0.00682 (19)	−0.00298 (17)	−0.00209 (18)
O2	0.0205 (2)	0.0129 (2)	0.0200 (2)	0.00460 (17)	0.00061 (18)	0.00015 (16)
O3	0.00783 (18)	0.0262 (3)	0.0214 (2)	−0.00199 (17)	0.00373 (16)	−0.00439 (19)
N1	0.0078 (2)	0.0195 (2)	0.0149 (2)	−0.00112 (17)	0.00092 (16)	0.00008 (18)
C1	0.0092 (2)	0.0114 (2)	0.0113 (2)	0.00061 (16)	0.00190 (16)	−0.00003 (16)
C2	0.0138 (2)	0.0137 (2)	0.0125 (2)	−0.00096 (19)	0.00073 (18)	−0.00101 (18)
C3	0.0212 (3)	0.0150 (3)	0.0140 (2)	−0.0010 (2)	0.0038 (2)	−0.00282 (19)
C4	0.0218 (3)	0.0166 (3)	0.0184 (3)	0.0025 (2)	0.0088 (2)	−0.0024 (2)
C5	0.0133 (2)	0.0177 (3)	0.0190 (3)	0.0033 (2)	0.0064 (2)	0.0004 (2)
C6	0.0093 (2)	0.0130 (2)	0.0130 (2)	0.00103 (17)	0.00242 (17)	0.00133 (17)
C7	0.0114 (2)	0.0143 (2)	0.0133 (2)	−0.00146 (18)	−0.00035 (18)	0.00154 (18)
C8	0.0115 (2)	0.0162 (2)	0.0113 (2)	−0.00114 (19)	0.00154 (18)	−0.00122 (18)

### Geometric parameters (Å, º)

**Table:**

S1—O2	1.4450 (6)	C2—H2	0.9500
S1—O3	1.4464 (6)	C3—C4	1.3960 (11)
S1—C1	1.7478 (6)	C3—H3	0.9500
S1—C8	1.7649 (7)	C4—C5	1.3898 (11)
O1—C7	1.2203 (8)	C4—H4	0.9500
N1—C7	1.3661 (9)	C5—C6	1.3982 (9)
N1—C6	1.4043 (9)	C5—H5	0.9500
N1—H1	0.890 (8)	C7—C8	1.5175 (9)
C1—C2	1.3945 (9)	C8—H8A	0.9900
C1—C6	1.4010 (9)	C8—H8B	0.9900
C2—C3	1.3892 (10)		
			
O2—S1—O3	117.64 (4)	C5—C4—C3	121.22 (6)
O2—S1—C1	109.21 (3)	C5—C4—H4	119.4
O3—S1—C1	109.56 (3)	C3—C4—H4	119.4
O2—S1—C8	108.59 (3)	C4—C5—C6	119.96 (6)
O3—S1—C8	109.59 (3)	C4—C5—H5	120.0
C1—S1—C8	100.95 (3)	C6—C5—H5	120.0
C7—N1—C6	127.24 (6)	C5—C6—C1	118.33 (6)
C7—N1—H1	116.1 (10)	C5—C6—N1	119.08 (6)
C6—N1—H1	116.7 (10)	C1—C6—N1	122.58 (6)
C2—C1—C6	121.72 (6)	O1—C7—N1	122.05 (6)
C2—C1—S1	119.61 (5)	O1—C7—C8	121.31 (6)
C6—C1—S1	118.58 (5)	N1—C7—C8	116.53 (6)
C3—C2—C1	119.34 (6)	C7—C8—S1	112.57 (4)
C3—C2—H2	120.3	C7—C8—H8A	109.1
C1—C2—H2	120.3	S1—C8—H8A	109.1
C2—C3—C4	119.38 (6)	C7—C8—H8B	109.1
C2—C3—H3	120.3	S1—C8—H8B	109.1
C4—C3—H3	120.3	H8A—C8—H8B	107.8
			
O2—S1—C1—C2	−93.69 (6)	C2—C1—C6—C5	−0.74 (9)
O3—S1—C1—C2	36.48 (6)	S1—C1—C6—C5	−177.28 (5)
C8—S1—C1—C2	152.03 (5)	C2—C1—C6—N1	178.32 (6)
O2—S1—C1—C6	82.92 (6)	S1—C1—C6—N1	1.78 (8)
O3—S1—C1—C6	−146.91 (5)	C7—N1—C6—C5	−165.40 (7)
C8—S1—C1—C6	−31.36 (6)	C7—N1—C6—C1	15.55 (10)
C6—C1—C2—C3	−1.16 (10)	C6—N1—C7—O1	−176.50 (7)
S1—C1—C2—C3	175.34 (5)	C6—N1—C7—C8	7.37 (10)
C1—C2—C3—C4	1.92 (10)	O1—C7—C8—S1	141.26 (6)
C2—C3—C4—C5	−0.81 (11)	N1—C7—C8—S1	−42.58 (7)
C3—C4—C5—C6	−1.12 (11)	O2—S1—C8—C7	−64.45 (5)
C4—C5—C6—C1	1.86 (10)	O3—S1—C8—C7	165.81 (5)
C4—C5—C6—N1	−177.23 (6)	C1—S1—C8—C7	50.29 (5)

### Hydrogen-bond geometry (Å, º)

*Cg*2 is the centroid of the C1–C6 benzene ring.

**Table:**

*D*—H···*A*	*D*—H	H···*A*	*D*···*A*	*D*—H···*A*
N1—H1···O3^i^	0.89 (1)	2.08 (1)	2.9472 (8)	165 (1)
C5—H5···O1^ii^	0.95	2.58	3.2330 (9)	126
C8—H8*A*···*Cg*2^iii^	0.99	2.93	3.7933 (8)	146
C8—H8*B*···O2^iv^	0.99	2.39	3.2126 (9)	140

Symmetry codes: (i) *x*+1, *y*, *z*; (ii) −*x*+3/2, *y*−1/2, −*z*+3/2; (iii) −*x*+1/2, *y*+1/2, −*z*+3/2; (iv) −*x*+1/2, *y*−1/2, −*z*+3/2.
